# Myocardial tissue characterization and strain analysis in healthy pregnant women using cardiovascular magnetic resonance native T1 mapping and feature tracking technique

**DOI:** 10.1186/s12968-018-0476-5

**Published:** 2018-08-02

**Authors:** Masafumi Nii, Masaki Ishida, Kaoru Dohi, Hiroaki Tanaka, Eiji Kondo, Masaaki Ito, Hajime Sakuma, Tomoaki Ikeda

**Affiliations:** 1 Department of Obstetrics and Gynecology, Mie University Hospital, 2-174 Edobashi, Tsu, Mie 514-8507 Japan; 2 Department of Radiology, Mie University Hospital, 2-174 Edobashi, Tsu, Mie 514-8507 Japan; 3 Department of Cardiology and Nephrology, Mie University Hospital, 2-174 Edobashi, Tsu, Mie 514-8507 Japan

**Keywords:** Cardiovascular magnetic resonance, Cardiac function, Native myocardial T1 mapping, Pregnancy, Myocyte mass, Peripartum cardiomyopathy

## Abstract

**Background:**

Peripartum cardiomyopathy is a life-threatening condition that occurs during the peripartum period in previously healthy women. Cardiovascular magnetic resonance (CMR) T1 mapping permits sensitive detection of tissue edema and fibrosis, and it may be useful in identifying altered myocardial tissue characteristics in peripartum cardiomyopathy. However, left ventricular (LV) volumes and mass increase considerably even in normal pregnancy, and it is not known whether altered tissue characteristics can be found in normal pregnancy. The aim of this study was to investigate whether the LV remodeling observed in normal pregnancy is associated with altered tissue characteristics determined by CMR.

**Methods:**

Twelve normal pregnant women and 15 non pregnant women underwent cine CMR and myocardial T1 measurement at 1.5 T. Pregnant women were scanned three times, in the 2nd and 3rd trimesters of pregnancy and at 1 month postpartum. LV volumes, LV mass (LVM), and global longitudinal strain (GLS) were analyzed by cine CMR. Native myocardial T1 was determined using modified Look-Locker inversion recovery (MOLLI) images.

**Results:**

LV end-diastolic volume (EDV) was significantly greater in the 3rd trimester (126 ± 22 mL) than in non-pregnant women (108 ± 14 mL, *p* < 0.05). LVM was significantly greater in the 3rd trimester (88.7 ± 11.8 g) than at 1 month postpartum (70.0 ± 9.8 g, p < 0.05) and in non-pregnant women (66.3 ± 13.9 g, p < 0.05). Myocardial native T1 among the 2nd and 3rd trimesters, 1 month postpartum, and non-pregnant women were similar (1133 ± 55 ms, 1138 ± 86 ms, 1105 ± 45 ms, and 1129 ± 52 ms, respectively, *p* = 0.59) as were GLS (− 19.5 ± 1.8, − 19.7% ± 2.2, − 19.0% ± 2.0%, and − 19.3% ± 1.9%, respectively, *p* = 0.66).

**Conclusions:**

LV remodeling during normal pregnancy is associated with myocardial hypertrophy, but not with edema or diffuse fibrosis of the myocardium or LV contractile dysfunction. These results observed in normal pregnancy will serve as an important basis for identifying myocardial abnormalities in patients with peripartum cardiomyopathy and other pregnancy-related myocardial diseases.

## Background

Peripartum cardiomyopathy (PPCM) is a life-threatening condition that occurs during the peripartum period in previously healthy women [1, 2]. In Western countries, 0.02–0.03% of pregnant women develop PPCM [1, 2]. The precise mechanism that leads to PPCM remains uncertain [3]. Moreover, the early recognition of cardiomyopathy remains elusive, and it often only becomes apparent when the woman is symptomatic and already has well-established disease. Early diagnosis by non-invasive imaging techniques would have considerable value because it would permit early interventions to prevent disease progression.

Cardiovascular magnetic resonance (CMR) can be used during the second or third trimester of pregnancy for both the mother and fetus because no adverse effects of CMR during those periods for both mother and fetus have been reported in the literature [4–6]. CMR can provide a non-invasive assessment of cardiac structure, function, and tissue characteristics without the limitations imposed by variations in ventricular geometry or exposure to ionizing radiation. Cine CMR is a highly accurate and reproducible technique in the determination of cardiac volume and mass [7]. While late gadolinium enhancement (LGE) CMR is an effective and reproducible method of assessing focal myocardial fibrosis [8] and acute myocardial damage [9], LGE CMR requires administration of gadolinium contrast medium.

Native T1 mapping is a novel technique allowing quantitative assessment of diffuse myocardial tissue properties without use of gadolinium-based contrast medium [10]. Native T1 is influenced by the presence of edema, diffuse fibrosis, and protein deposition in the myocardium, showing increases in values [11]. Recognition of myocardial edema in acute myocarditis or Takotsubo cardiomyopathy by native T1 mapping was shown to be superior to that by T2-weighted sequences and LGE [12]. A prolonged native T1 in dilated cardiomyopathy patients correlates closely with histological fibrosis [13]. Native myocardial T1 values are increased in patients with various types of myocardial diseases, including dilated cardiomyopathy, hypertrophic cardiomyopathy, and aortic stenosis etc., reflecting the degree of diffuse myocardial fibrosis [14].

The prevalence of LGE in PPCM, which represents focal replacement fibrosis, varies substantially, ranging from 5 to 71% in the literature [15–19]. Thus, the utility of LGE CMR in clinical practice remains controversial. Native T1 mapping might be useful in the detection of PPCM in the early disease stage, since native T1 is a quantitative measure that can detect the subtle changes of diffuse myocardial fibrosis or edema.

Circulating blood volume increases by approximately 40% in normal pregnancy [20]. The increased blood volume in normal pregnancy leads to morphological and functional changes in the heart, such as increased left ventricular (LV) mass (LVM), [21–23] increased LV and atrial (LA) volumes, [21, 22] and reduced LV diastolic function [23]. Thus, in response to the major physiologic alterations in the maternal cardiovascular system throughout pregnancy, reversible morphological alterations, known as cardiac remodeling, are provoked in the maternal heart, ensuring a normotensive course of pregnancy [4, 7, 24]. The drastic changes in heart morphology and function in normal pregnancy may be associated with alterations of myocardial tissue characteristics. Therefore, CMR, especially T1 mapping, might have great potential in the management of PPCM and other pregnancy-related myocardial diseases. However, it is essential to understand the reference values of CMR parameters, including native T1, in normal pregnancy to identify myocardial abnormalities in patients with PPCM and other pregnancy-related myocardial diseases.

Consequently, the aim of this study was to investigate whether the LV remodeling observed in normal pregnancy is associated with altered tissue characteristics determined by CMR.

## Methods

This prospective study was conducted in accordance with the principles of the Declaration of Helsinki and with the approval of the Institutional Review Board (2858, February 5, 2014). All women gave their written, informed consent prior to participation in this study.

### Participants

Fourteen healthy pregnant women were prospectively recruited from between June 2015 and August 2016. Eligible subjects were between the age of 20 and 39 years at their last normal menstrual period, carrying a healthy singleton pregnancy. Exclusion criteria included any history of hypertension, diabetes mellitus, smoking, thyroid disease, known cardiac disease, any pregnancy-related complications including preeclampsia, hypertensive disorders of pregnancy, gestational diabetes, and the presence of a pacemaker or general contraindications to CMR. Of the 14 pregnant women, two were subsequently excluded because of hypertensive disorders of pregnancy. All 12 remaining women (33 ± 4 years) had a normal delivery between September 2015 and November 2016. This population was compared with 15 age-matched healthy non-pregnant women (31 ± 3 years). All 12 pregnant women underwent CMR three times, in the 2nd and 3rd trimesters of pregnancy and at 1 month postpartum, with a simultaneous 5.0 mL blood sample drawn to determine concentrations of hemoglobin (Hb) and brain type natriuretic peptide (BNP). Fifteen non-pregnant women also underwent CMR and provided a 5.0 mL blood sample immediately after the CMR examination and served as control women.

### CMR image acquisition

CMR studies were performed on a 1.5 T CMR scanner (Achieva 1.5 T, Philips Healthcare, Best, The Netherlands) using a body coil for signal reception. No sedative medications or contrast agents were used, and all participants were imaged in the half left lateral decubitus position to minimize aortocaval compression. The CMR study protocol included cine CMR and native T1 mapping using a modified Look-Locker inversion recovery (MOLLI) sequence. Cine MR images were acquired with a retrospective electrocardiographic gating and a segmented steady-state-free precession sequence during brief periods of breath-holding at a shallow expiration in the following planes: LV 2-chamber and 4-chamber views and short-axis planes covering the entire LV and right ventricle (RV) (repetition time [TR] 2.9 ms; echo time [TE] 1.44 ms; flip angle [FA] 55°; field of view [FOV] 350 × 350 mm^2^; acquisition matrix 176 × 176; reconstruction matrix 256 × 256; slice thickness 10 mm). All cine images were acquired with 20 phases per cardiac cycle. The short-axis plane was defined as the perpendicular plane to the horizontal and vertical long-axis views. Single-slice native T1 mapping was performed using a 17-heartbeat steady-state-free procession MOLLI sequence on the short-axis imaging plane at the level of the mid LV (TR, 2.3 ms; TE, 0.83 ms; FA, 35°; FOV, 300 × 327 mm^2^; acquisition matrix, 176 × 140; reconstruction matrix, 288 × 288; slice thickness, 10 mm) [25].

### CMR image analysis

**Fig. 1:**
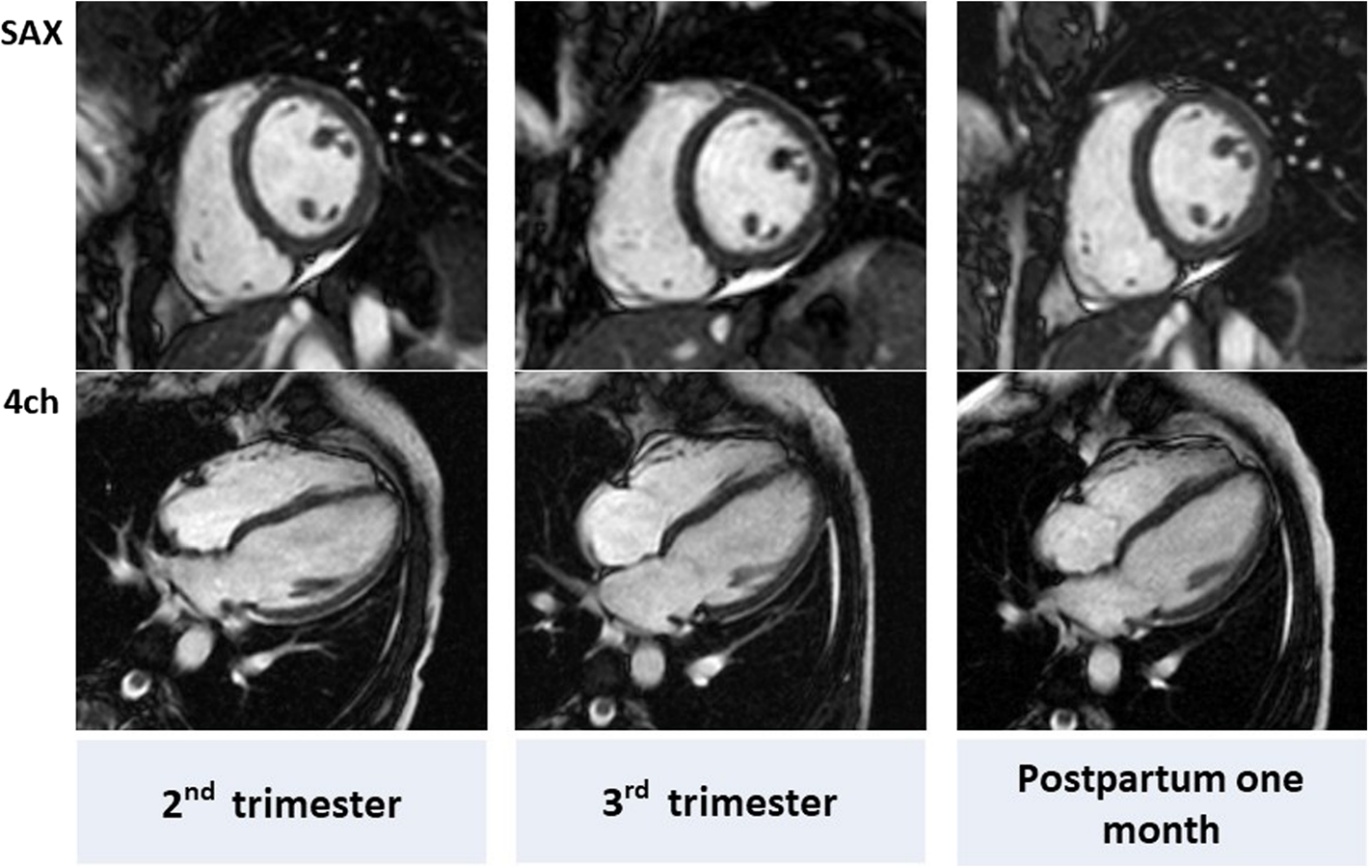
Cine CMR in a representative pregnant woman with end-diastolic volume (EDV), end-systolic volume (ESV), ejection fraction (EF) and left ventricular mass (LVM) of 132 mL, 69 mL, 48.0% and 106 g, respectively in the 2nd trimester, 136 mL, 60 mL, 55.8% and 95 g, respectively in the 3rd trimester, and 112 mL, 57 mL, 53.6% and 76 g, respectively at 1 month postpartum

**Fig. 2:**
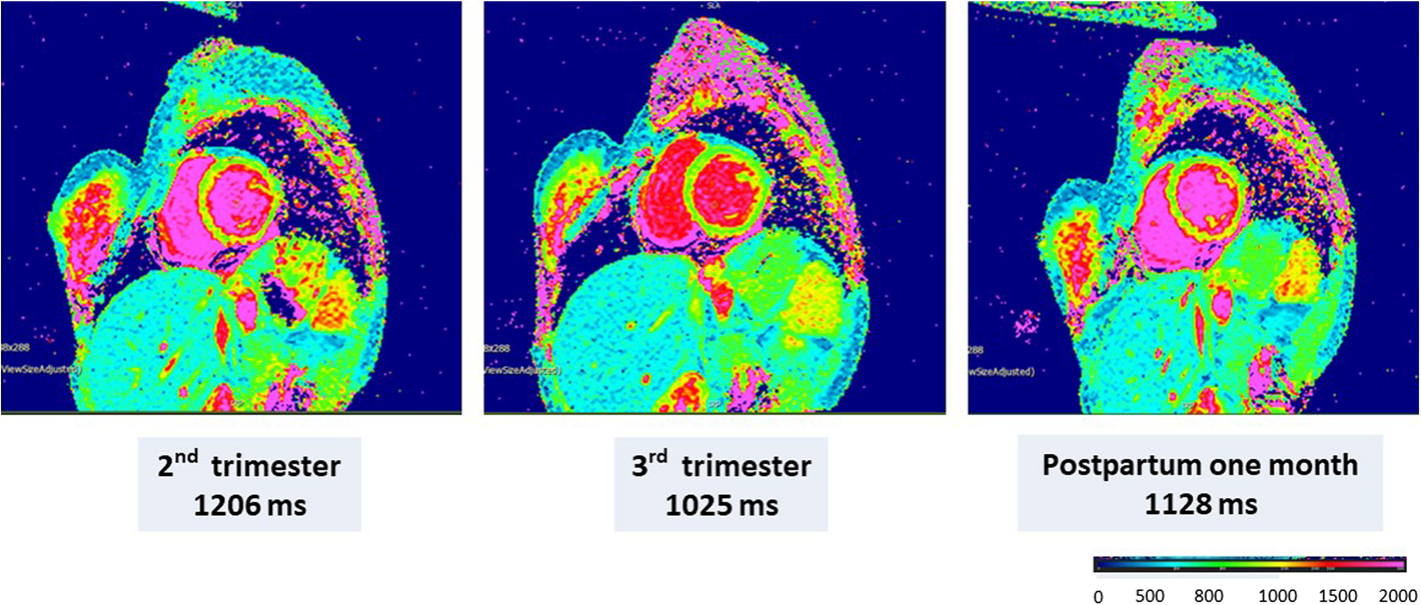
Native T1 mapping using MOLLI in a representative pregnant woman, with a native T1 of 1206 ms in the 2nd trimester, 1025 ms in the 3rd trimester, and 1128 ms at 1 month postpartum

CMR image analyses were carried out by an expert observer who had 15 years of CMR experience (M.I.) using CMR analysis software, cvi42 (Circle Cardiovascular Imaging Inc., Calgary, Canada). LV volume and function were analyzed based on the short-axis cine stack (Fig. 1). The endocardial and epicardial borders of the LV wall were manually traced on cine CMR images in end-diastolic and end-systolic phases. LVM was calculated as the volume of the LV myocardium multiplied by the specific gravity of the myocardium (1.05 g/ml) [26]. Mean LV wall thickness was measured in the end-diastolic phase on the short-axis view. The papillary muscles and trabeculations were not included in the myocardial mass calculation. Then, RV volume and function were analyzed based on the short-axis cine stack. LV and RV measurements were indexed to body surface area (BSA). Strain analysis was performed by a feature-tracking algorithm [11]. The endocardial and epicardial borders of LV and RV myocardium were manually traced in the end-diastolic phase of a 4-chamber view cine CMR image. Then, the software automatically propagated the endocardial and epicardial contours and tracked the motion of the in-plane tissue voxels through the entire cardiac cycle. Consequently, peak longitudinal strain values were recorded at global levels for the LV and RV. Native T1 measurement was performed pixel-wise. After correcting for respiratory motion of the images by non-rigid image registration, T1 maps were generated by fitting pixels to the equation s (t) = a-b exp. (−t/T1*), and T1 = (b/a-1) T1*, where a and b are constants, t is time, and s (t) is signal intensity at time t [27]. The resulting pixel-wise native T1 maps were stored in DICOM format, and native T1 values were averaged for all pixels (Fig. 2).

### Statistical methods

The statistical analyses were performed using SPSS Statistics 20.0 (International Business Machiens, Armonk, New York, USA). Data were normally distributed (Kolmogorov-Smirnov test), and all data are therefore presented as means ± standard deviation (SD). Changes in variables within a group were compared using the paired *t*-test with the post hoc Tukey-Kramer method. Analysis of variance (ANOVA) was used for comparisons of multiple groups. Pearson’s product-moment correlation coefficient was used to measure linear correlations between two variables. In all analyses, *p* < 0.05 was taken to indicate significance. However, a significant linear correlation between two variables was defined as that meeting both *p* < 0.05 and correlation coefficient (r-value) > 0.2 or < − 0.2 [28].

## Results

### Demographic characteristics of participants

The demographic characteristics of the healthy pregnant women and non-pregnant women are summarized in Table 1. Between pregnant and non-pregnant women were similar in age (33 ± 4 and 31 ± 3 years, *p* = 0.126), body mass index (BMI) (19.3 ± 0.7 and 20.2 ± 1.5 kg/m^2^, *p* = 0.067), and BSA (1.50 ± 0.09 and 1.49 ± 0.09 m^2^, *p* = 0.848). However, the proportion of nulliparous women was significantly different (5 (42%) and 14 (93%) respectively, p < 0.05, Fisher’s exact test).

**Table 1:** Demographic characteristics of the participating women

	Pregnant	Non-pregnant
Number of women	12	15
Number of nulliparous women	5 (42%)†	14 (93%)
Age (y)	33 ± 4	31 ± 3
Pre-pregnancy BMI (kg/m^2^)	19.3 ± 0.7	20.2 ± 1.5
Pre-pregnancy BSA (m^2^)	1.50 ± 0.09	1.49 ± 0.09
Pre-pregnancy Hb (g/dL)	NA	13.0 ± 0.9
Pre-pregnancy BNP (pg/mL)	NA	13 ± 5
BSA (m^2^) at CMR		
2nd trimester	1.53 ± 0.10	NA
3rd trimester	1.57 ± 0.11	NA
Postpartum 1 month (PP1M)	1.52 ± 0.11	NA
Hb (g/dL) at CMR		
2nd trimester	11.2 ± 1.0 *†	NA
3rd trimester	11.2 ± 0.8*†	NA
Postpartum 1 month (PP1M)	12.3 ± 1.0	NA
BNP (pg/mL) at CMR		
2nd trimester	20 ± 1 *†	NA
3rd trimester	14 ± 6	NA
Postpartum 1 month (PP1M)	11 ± 8	NA
Gestational week at delivery	39.3 ± 1.4	NA

*BMI* Body mass index, *BSA* Body surface area, *CMR* Cardiovascular magnetic resonance,

*[Hb]/[BNP]* Blood concentration of hemoglobin/brain type natriuretic peptide, *NA* Not applicable

*, *P* < 0.05 vs. postpartum 1 month; †, *P* < 0.05 vs. non-pregnant women

CMR examination and blood tests were performed at gestational week 25.3 ± 0.6 (26.4 to 24.0) and 33.7 ± 0.7 (34.9 to 33.1) for the 2nd and 3rd trimesters of pregnancy, respectively, and on postpartum day 35 ± 4 (41 to 31).

Hemoglobin concentrations were significantly higher 1 month postpartum than in the 2nd, 3rd trimesters and in non-pregnant women. The BNP concentration was significantly higher in the 2nd trimester than at 1 month postpartum and in non-pregnant women.

### LV and RV parameters on cine CMR

**Fig. 3:**
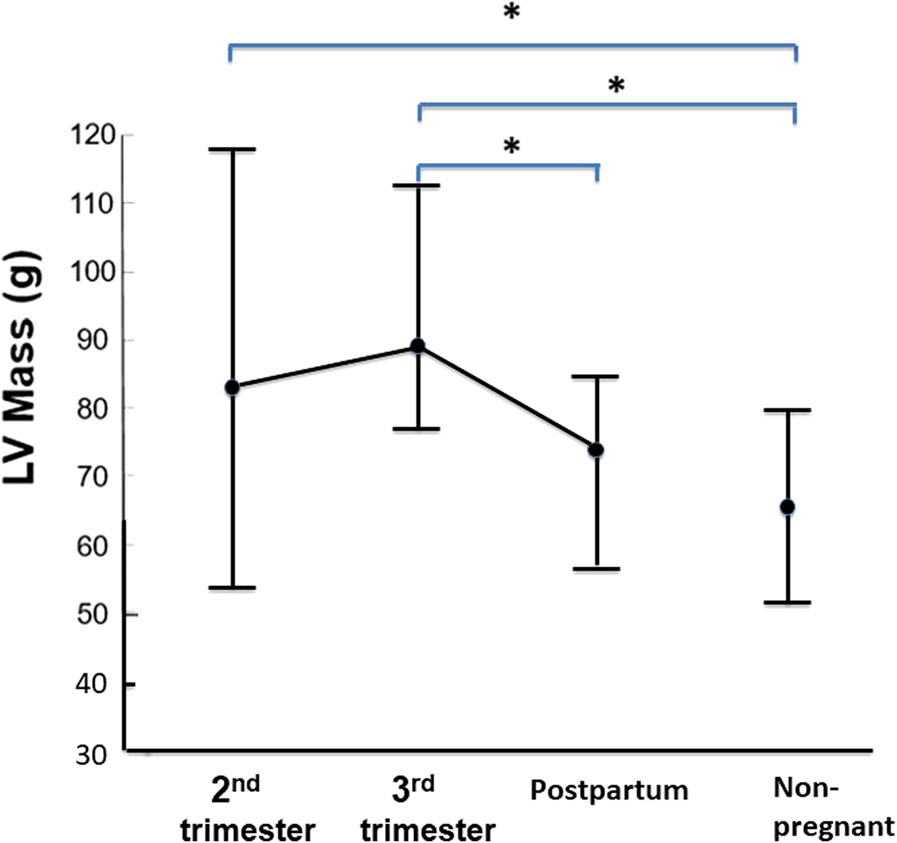
LVM by the stages of pregnancy in healthy pregnant women and in non-pregnant women. Mean LVM is 82.6 ± 18.2 g in the 2nd trimester, 88.7 ± 11.8 g in the 3rd trimester, 72.0 ± 9.8 g at 1 month postpartum, and 66.3 ± 13.9 g in non-pregnant women. *, *P* < 0.05

The structural and systolic functional parameters of the LV and RV determined by cine CMR, including peak global longitudinal strain (GLS), in the pregnant women and in the non-pregnant women are summarized in Tables 2 and 3, respectively. In pregnant women, LV stroke volume (SV) was not substantially altered during pregnancy compared to 1 month postpartum, but heart rate (HR) was significantly higher in the 2nd trimester than at 1 month postpartum (76 ± 8 vs 64 ± 8 bpm; *p* < 0.05), resulting in significantly greater cardiac output (CO) and cardiac index (CI) in the 2nd trimester (5.7 ± 1.6 L/min and 3.7 ± 0.9 L/min/m^2^) than at 1 month postpartum (4.3 ± 1.1 L/min and 2.8 ± 0.7 L/min/m^2^, *p* < 0.05) and in non-pregnant women (4.3 ± 0.7 L/min and 2.9 ± 0.4 L/min/m^2^, *p* < 0.05), respectively. As with LV, RV CO (6.2 ± 1.3 mL/min) and CI (4.0 ± 0.7 mL/min/m^2^) were significantly greater in the 2nd trimester than at 1 month postpartum (4.9 ± 0.9 mL/min, *p* < 0.05 and 3.2 ± 0.5 mL/min/m^2^, p < 0.05) and in non-pregnant women (4.8 ± 0.6 mL/min, *P* < 0.05 and 3.2 ± 0.3 mL/min/m^2^, p < 0.05), respectively. LV end-diastolic volume (EDV) was significantly greater in the 3rd trimester (126 ± 22 mL) than in non-pregnant women (108 ± 14 mL, p < 0.05). LVM and LVM index were significantly greater in the 3rd trimester (88.7 ± 11.8 g and 56.5 ± 6.5 g/m^2^) than at 1 month postpartum (72.0 ± 9.8 g, p < 0.05 and 47.5 ± 6.0 g/m^2^, p < 0.05) and in non-pregnant women (66.3 ± 13.9 g, p < 0.05 and 44.5 ± 8.0 g/m^2^, p < 0.05), respectively (Fig. 3). Mean LV wall thickness was also significantly greater in the 3rd trimester (5.09 ± 0.57 mm) than at 1 month postpartum (4.53 ± 0.50 mm, p < 0.05) and in non-pregnant women (4.57 ± 0.63 g, p < 0.05). Pearson’s linear correlation coefficients were 0.76 (*p* < 0.001), 0.75 (p < 0.001), and 0.56 (p < 0.001) for LVEDV, LV end-systolic volume (ESV), and LVM, respectively, against actual body weight.

**Table 2:** Left ventricular parameters on CMR

	Pregnant women			
	2nd (*n* = 12)	3rd (*n* = 12)	Postpartum (*n* = 12)	Non-pregnant women (*n* = 15)
LVSV (mL)	74.0 ± 18.1†	71.1 ± 13.7	66.0 ± 13.1	62.2 ± 9.8
LVSVI (mL/m^2^)	48.1 ± 10.4	45.3 ± 7.6	43.4 ± 7.9	41.7 ± 6.5
LVCO (L/min)	5.7 ± 1.6*†	5.2 ± 1.2†	4.3 ± 1.1	4.3 ± 0.7
LVCI (L/min/m^2^)	3.7 ± 0.9*†	3.3 ± 0.7	2.8 ± 0.7	2.9 ± 0.4
LVEF (%)	60.2 ± 8.7	56.7 ± 5.0	57.7 ± 4.5	57.4 ± 4.6
LVEDV (mL)	123 ± 21	126 ± 22†	115 ± 21	108 ± 14
LVEDVI (mL/m^2^)	79.8 ± 9.8	80.0 ± 11.1	75.0 ± 10.4	73.2 ± 9.3
LVESV (mL)	48.7 ± 13.5	54.5 ± 11.5†	48.6 ± 10.4	46.4 ± 8.3
LVESVI (mL/ m^2^)	31.7 ± 8.0	34.6 ± 5.8	31.7 ± 4.9	31.4 ± 5.3
LVM (g)	82.6 ± 18.2†	88.7 ± 11.8*†	72.0 ± 9.8	66.3 ± 13.9
LVMI (g/m^2^)	53.7 ± 10.2†	56.5 ± 6.5*†	47.5 ± 6.0	44.5 ± 8.0
LVM/EDV (g/mL)	0.68 ± 0.13	0.72 ± 0.13	0.65 ± 0.14	0.61 ± 0.14
Heart rate (bpm)	76 ± 8*†	73 ± 7*	64 ± 8	69 ± 9
LV peak strain (%)	−19.5 ± 1.8	−19.7 ± 2.2	−19.0 ± 2.0	−19.3 ± 1.9
LV mean wall thickness (mm)	4.8 ± 0.7	5.1 ± 0.5*†	4.5 ± 0.5	4.6 ± 0.6
LV mean wall thickness index (mm/m^2^)	3.1 ± 0.4	3.2 ± 0.3	3.0 ± 0.3	3.0 ± 0.4

*2nd and 3rd* Trimesters of pregnancy, *BSA* Body surface area;

*LV* Left ventricular, *LVM* LV mass, *SV* Stroke volume, *CO* Cardiac output, *EF* Ejection fraction,

*EDV* End-diastolic volume, *ESV* End-systolic volume

*, *P* < 0.05 vs. data for postpartum one month; †, *P* < 0.05 vs. data for non-pregnant women

**Table 3:** Right ventricular parameters on CMR

	Pregnant women (*n* = 12)			
	2nd (*n* = 12)	3rd (*n* = 12)	Postpartum (*n* = 12)	Non-pregnant women (*n* = 15)
RVSV (mL)	80.8 ± 13.5	78.8 ± 12.2	75.8. ± 13.1	71.1 ± 10.3
RVSVI (mL/m^2^)	52.6 ± 6.8	50.5 ± 4.7	49.7 ± 6.8	47.5 ± 5.4
RVCO (L/min)	6.2 ± 1.3*†	5.7 ± 1.1†	4.9 ± 0.9	4.8 ± 0.6
RVCI (L/min/m^2^)	4.0 ± 0.7*†	3.6 ± 0.6†	3.2 ± 0.5	3.2 ± 0.3
RVEF (%)	60.3 ± 3.8†	60.2 ± 3.9†	59.3 ± 6.3	56.2 ± 5.2
RVEDV (mL)	133 ± 19	129 ± 18	126 ± 20	126 ± 18
RVEDVI (mL/m^2^)	86.3 ± 9.7	82.2 ± 8.3	82.9 ± 10.2	84.2 ± 9.5
RVESV (mL)	53.3 ± 9.7	52.3 ± 10.0	50.5 ± 11.4	55.0 ± 12.5
RLVESVI (mL/ m^2^)	34.7 ± 5.3	33.3 ± 5.5	33.2 ± 6.7	36.8 ± 7.8
RV peak strain (%)	−28.0 ± 3.1	−25.6 ± 3.4	−25.7 ± 3.9	−26.0 ± 5.0

*2nd and 3rd* Trimesters of pregnancy, *BSA* Body surface area,

*RV* Right ventricular, *SV* Stroke volume, *CO* Cardiac output, *EF* Ejection fraction,

*EDV* End-diastolic volume, *ESV* End-systolic volume; *, *P* < 0.05 vs. data for postpartum 1 month;

†, *P* < 0.05 vs. data for non-pregnant women

### LV strain by feature tracking

**Fig. 4:**
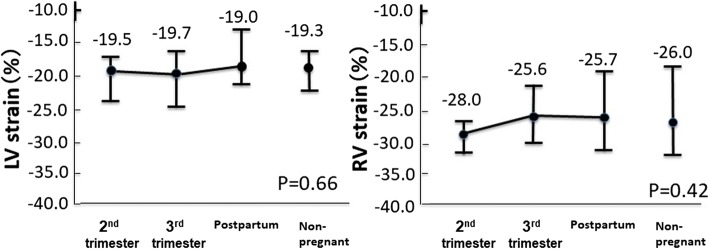
Peak global longitudinal strain (GLS) (%) in the LV and right ventricle (RV) in pregnant women and non-pregnant women. Neither the LV nor the RV GLS shows significant differences among the 2nd and 3rd trimesters, 1 month postpartum, and non-pregnant women

LV GLS showed no significant difference among the 2nd and 3rd trimesters, 1 month postpartum, and in non-pregnant women (− 19.5 ± 1.8, − 19.7% ± 2.2, − 19.0% ± 2.0%, and − 19.3 ± 1.9%, respectively, *p* = 0.66) (Fig. 4). RV GLS also showed no significant difference among the 2nd and 3rd trimesters, 1 month postpartum, and non-pregnant women (− 28.0 ± 3.1, − 25.6% ± 3.4, − 25.7% ± 3.9%, and − 26.0% ± 5.0%, respectively, *p* = 0.42) (Fig. 4).

### Native myocardial T1 determined by the MOLLI sequence

**Fig. 5:**
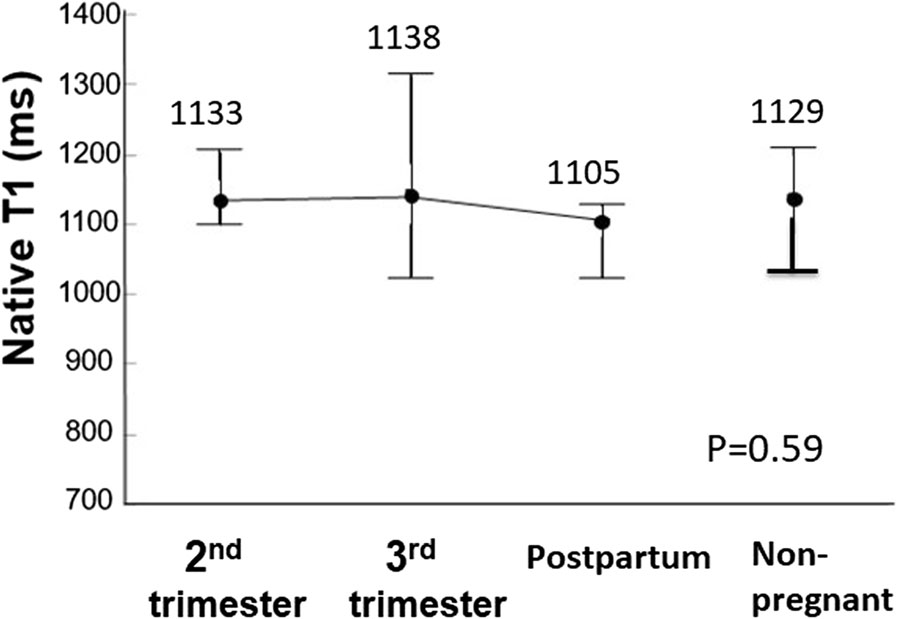
Native T1 determined using MOLLI. Change in the native T1 using MOLLI by the stage of pregnancy and in non-pregnant women. Native T1 shows no significant differences among the 2nd and 3rd trimesters, 1 month postpartum, and non-pregnant women

**Fig. 6:**
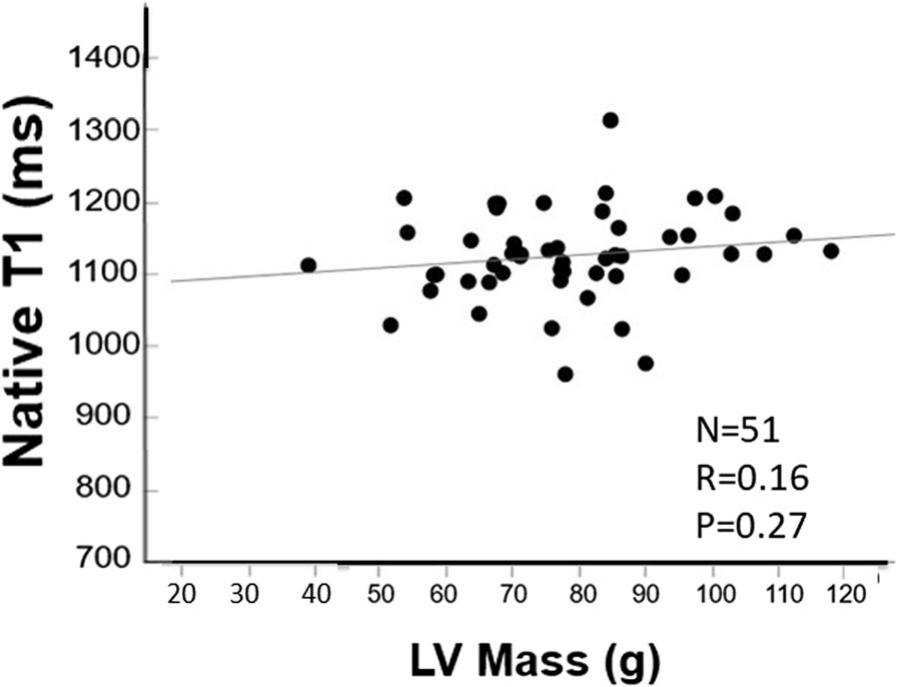
Association between LVM and native T1 during pregnancy and in non-pregnant women. N = number of samples, R = correlation efficient, P=*P* value

Native myocardial T1 in pregnant women was 1133 ± 55 ms (95%CI 1095.8 to 1169.4 ms), 1138 ± 86 ms (95%CI 1081 to 1194 ms), and 1105 ± 45 ms (95% CI 1075 to 1134 ms) in the 2nd trimester, the 3rd trimester, and at 1 month postpartum, respectively, whereas native myocardial T1 in non-pregnant women was 1129 ± 52 ms (95% CI 1100 to 1159 ms) (Fig. 5). Thus, no significant difference was noted in native myocardial T1 among the 2nd and 3rd trimesters, 1 month postpartum, and non-pregnant women (*p* = 0.59). Pearson’s linear correlation analysis demonstrated that native myocardial T1 was not correlated with LVM, with r of 0.16 and *p* = 0.27 (Fig. 6).

## Discussion

In this study, reference values of CMR parameters including native myocardial T1 and LV GLS in normal pregnancy were determined in 12 Japanese normal pregnant women. It was found that, in normal pregnancy, LV remodeling occurs without significant alterations of native myocardial T1 and LV GLS, despite the significant increase in LVM. These results observed in normal pregnancy will serve as an important basis for identifying myocardial abnormalities in patients with PPCM and other pregnancy-related myocardial diseases.

LVM increased significantly during pregnancy in the normotensive pregnant women in the current study. This finding is consistent with the results in previous studies using echocardiography and CMR [4, 21–23]. In the present study, native T1 in healthy pregnant women showed no significant changes throughout pregnancy and postpartum and as compared with non-pregnant women. The finding that native T1 in normal pregnancy is comparable to that in non-pregnant women confirmed that measurement of native T1 can be as useful to diagnose cardiomyopathy or myocarditis in normal pregnancy as in non-pregnant patients [14]. This finding also suggests that interstitial water retention does not occur in the myocardium of uncomplicated pregnant women.

The myocardium consists of cellular and extracellular interstitial compartments. Therefore, increased LVM without myocardial edema in normal pregnancy in the present study is attributable to an increase in cardiomyocyte volume or increase of the intravascular compartment. Eghbali et al. demonstrated in their animal study that mouse cardiomyocyte volume increased by approximately 70% in pregnancy [29]. In athletes’ hearts, which show the cardiac adaptive response to regular athletic training, cardiac adaptation is characterized by an increase in LVM and, as a consequence, eccentric hypertrophy [30]. As in the case of increased LVM in normal pregnant women that was observed in the present study, McDiarmid et al. recently showed, using CMR T1 mapping technique, that increased LVM in athletes’ hearts occurs because of an expansion of the cellular compartment rather than of extracellular volume [30]. However, blood volume alteration has not been observed in pregnancy-related physiological heart hypertrophy in mice [24, 29, 30].

In the current study, LV remodeling in healthy pregnant women was not associated with impairment of LV GLS. LV hypertrophy is observed in a variety of LV myocardial conditions such as hypertension, aortic stenosis, hypertrophic cardiomyopathy, amyloidosis, Fabry disease, etc., [31] and in patients with heart failure with preserved ejection fraction [32]. A recent study demonstrated that LV hypertrophy in Fabry disease was associated with significant impairment of LV GLS, even though LV ejection fraction was preserved [33]. Another study showed that GLS is impaired in patients with heart failure with preserved ejection fraction (HFpEF) [34]. The present results suggest that increased LVM in healthy pregnant women is the adaptive response to an increased heart workload.

Significant positive correlations between body weight and LV volume and LVM were observed in the present study. It is known that LV volume and LVM increase along with the increase of body weight even in non-pregnant women [35]. However, pregnancy-related cardiac remodeling can be a complex process that involves many factors, including changes in the signaling pathways and composition of extracellular matrix, as well as the levels of sex hormones. Underlying molecular mechanisms of cardiac remodeling during human pregnancy remain unknown. In mouse experiments, pregnancy-related physiologic heart hypertrophy was different from pathologic hypertrophy in terms of gene expression. It is supposed that the increase in estrogen toward the end of pregnancy plays a substantial role in the expressions of certain genes, which contributes to pregnancy-related heart hypertrophy [29]. Furthermore, some animal studies reported that fibrosis is minimal or absent in the pregnant heart in the rat [24]. The results of the current study are in line with these findings of previous animal studies [24, 29]. Thus, LV remodeling may not simply be attributable to the overweight and the natural volume overload during pregnancy.

The second and third trimesters of pregnancy and 1 month postpartum were selected to observe the time course of the CMR parameters. The first trimester was omitted because performing CMR in this period is still controversial in normal pregnant women. The reason why 24–28 weeks was selected in the 2nd trimester is that circulating blood volume starts to increase in the 1^st^ trimester and reaches almost its peak during this period [7, 36, 37]. The period of 32–36 weeks was selected in the 3rd trimester because circulating blood volume maintains its peak, and the heart overload is the greatest in this period [7, 36, 37]. Postpartum 3 months would be preferred to confirm that CMR parameters return to the baseline because the decrease in cardiac output toward baseline typically occurs between 6 and 12 weeks postpartum [7, 38]. However, it is almost impossible in Japan to obtain consent from all participants to perform CMR examinations or laboratory tests other than at postpartum 1 month, because all women and neonates in Japan see a doctor at postpartum 1 month as a routine visit. Therefore, 1 month postpartum was selected.

Early diagnosis of PPCM is important for better outcomes, because delay in the diagnosis of PPCM is associated with worse outcomes, such as death or heart transplantation [2, 39]. Myocardial tissue characterization by CMR in patients with PPCM has been only sporadically described, mainly using LGE CMR. However, the prevalence of LGE in PPCM varies substantially, ranging from 5 to 71% in those previous studies [15–19]. Although LGE CMR is a robust technique for identifying focally abnormal regions such as myocardial damage and fibrosis, LGE CMR is not sufficiently sensitive to detect diffuse myocardial disease. Furthermore, LGE CMR requires the administration of gadolinium contrast medium, which is recommended to be avoided until after delivery unless absolutely necessary [40]. Therefore, native T1 mapping, which is obtained without gadolinium contrast injection, may play an important role for the diagnosis of PPCM and the clinical management of PPCM patients.

In the present study, RVEDV showed an increasing trend during pregnancy. RV parameters are more easily accessible by CMR than by echocardiography, since CMR is not limited by variations of ventricular geometry, body habitus etc., which are major limiting factors of echocardiography. A previous study by Ducas et al. demonstrated that RVEDV was significantly increased from baseline during pregnancy [4]. The present findings are in accordance with the result from the previous study. More importantly, RVEF and RV GLS during pregnancy were not significantly altered compared with those at 1 month postpartum in the current study. This might represent the adaptive response of the RV to the increased heart workload, as in the LV. In the current study, some deviation was noted in LVEF and RVEF, and the standard deviations for LVEF were slightly higher than those for RVEF. However, they might occur because, even in the study of reference ranges for CMR in Korean population cohort, such differences were observed in male [41]. The considerably large standard deviation values for LVESV compared to the mean LVESV (i.e. 48.7 ± 13.5 mL) might be within the same range for LVESV as in the normal Chinese population (60 ± 12 ml) [42].

### Clinical implications

In this study, reference values of myocardial T1 relaxation time and GLS were determined in 12 Japanese normal pregnant women. These data are useful to determine if these CMR parameters are abnormal in patients with suspected PPCM. The current results can also serve as an important baseline not only in PPCM patients, but also in other pregnancy-related cardiac diseases, for example, in identifying myocardial edema or inflammation in pregnant patients with acute myocarditis.

### Limitations

There are three limitations in this study. First, there was a small number of participants. The age distribution of the pregnant women is an important issue since it pertains to the age range of 20–35 years and the age range of 35–39 years. In the present study population, the numbers of subjects in the age range of 20–35 years and the age range of 35–39 years were 8 and 4 in pregnant women and 12 and 3 in non-pregnant controls, respectively. However, because of the small sample size, it was not possible to show any significant age-related differences among the group and between groups. Future studies with a larger number of participants are warranted. Second, there was a difference in parity between pregnant women and non-pregnant controls. Third, no CMR findings were compared to the results of cardiac biopsy or heart catheterization as the gold standard technique for tissue characterization or functional assessment of the heart.

## Conclusion

Despite the presence of LV hypertrophy during pregnancy, T1 relaxation time measured by the CMR MOLLI method and myocardial strain by feature-tracking CMR were not different from those in non-pregnant women. These results observed in normal pregnancy will serve as an important basis for identifying myocardial abnormalities in patients with PPCM and other pregnancy-related myocardial diseases.

## Abbreviations

BMI: Body mass index
BNP: Brain type natriuretic peptide
BSA: Body surface area
CI: Cardiac index
CMR: Cardiovascular magnetic resonance
CO: Cardiac output
DICOM: Digital Imaging and Communication in Medicine
EDV: End-diastolic volume
EF: Ejection fraction
ESV: End-systolic volume
FA: Flip angle
FOV: Field of view
GLS: Global longitudinal strain
Hb: Hemoglobin
HFpEF: Heart failure with preserved ejection fraction
HR: Heart rate
LA: Left atrium/left atrial
LGE: Late gadolinium enhancement
LV: Left ventricle/left ventricular
LVM: Left ventricular mass
MOLLI: Modified Look Locker inversion recovery
PPCM: Peripartum cardiomyopathy
RV: Right ventricle/right ventricular
SV: Stroke volume
TE: Echo time
TR: Repetition time

## References

[1] Ersbøll AS, Damm P, Gustafsson F, Vejlstrup NG, Johansen M (2016). Peripartum cardiomyopathy: a systematic literature review. Acta Obstet Gynecol Scand.

[2] Kamiya CA, Yoshimatsu J, Ikeda T (2016). Peripartum cardiomyopathy from a genetic perspective. Circ J.

[3] Sliwa K, Hilfiker-Kleiner D, Petrie MC, Mebazaa A, Pieske B, Buchmann E (2010). Heart failure Association of the European Society of cardiology working group on Peripartum cardiomyopathy. Current state of knowledge on aetiology, diagnosis, management, and therapy of peripartum cardiomyopathy: a position statement from the heart failure Association of the European Society of cardiology working group on peripartum cardiomyopathy. Eur J Heart Fail.

[4] Ducas RA, Elliott JE, Melnyk SF, Premecz S, daSilva M, Cleverley K (2014). Cardiovascular magnetic resonance in pregnancy: insights from the cardiac hemodynamic imaging and remodeling in pregnancy (CHIRP) study. J Cardiovasc Magn Reson.

[5] Kanal E, Barkovich AJ, Bell C, Borgstede JP, Bradley WG, Froelich JW (2007). ACR blue ribbon panel on MR safety. ACR guidance document for safe MR practices: 2007. ARJ Am J Roentgenol.

[6] ACOG Committee Opinion (2004). Number 299, September 2004 (replaces no. 158, September 1995). Guidelines for diagnostic imaging during pregnancy. Obstet Gynecol.

[7] Stewart RD, Nelson DB, Matulevicius SA, Morgan JL, McIntire DD, Drazner MH (2016). Cardiac magnetic resonance imaging to assess the impact of maternal habitus on cardiac remodeling during pregnancy. Am J Obstet Gynecol.

[8] Amano Y, Takeda M, Tachi M, Kitamura M, Kumita S (2014). Myocardial fibrosis evaluated by look-locker and late gadolinium enhancement magnetic resonance imaging in apical hypertrophic cardiomyopathy: association with ventricular tachyarrhythmia and risk factors. J Magn Reson Imaging.

[9] Wong TC, Piehler KM, Zareba KM, Lin K, Phrampus A, Patel A (2013). Myocardial damage detected by late gadolinium enhancement cardiovascular magnetic resonance is associated with subsequent hospitalization for heart failure. J Am Heart Assoc.

[10] Cannaò PM, Altabella L, Petrini M, Alì M, Secchi F, Sardanelli F (2016). Novel cardiac magnetic resonance biomarkers: native T1 and extracellular volume myocardial mapping. Eur Heart J Suppl.

[11] Moon JC, Messroghli DR, Kellman P, Piechnik SK, Robson MD, Ugander M (2013). Society for Cardiovascular Magnetic Resonance Imaging; cardiovascular magnetic resonance working Group of the European Society of cardiology. Myocardial T1 mapping and extracellular volume quantification: a Society for Cardiovascular Magnetic Resonance (SCMR) and CMR working Group of the European Society of cardiology consensus statement. J Cardiovasc Magn Reson.

[12] Ferreira VM, Piechnik SK, Dall’Armellina E, Karamitsos TD, Francis JM, Ntusi N (2013). T(1) mapping for the diagnosis of acute myocarditis using CMR: comparison to T2-weighted and late gadolinium enhanced imaging. JACC Cardiovasc Imaging.

[13] Goto Y, Ishida M, Takase S, Sigfridsson A, Uno M, Nagata M (2017). Comparison of displacement encoding with stimulated echoes to magnetic resonance feature tracking for the assessment of myocardial strain in patients with acute myocardial infarction. Am J Cardiol.

[14] Haaf P, Garg P, Messroghli DR, Broadbent DA, Greenwood JP, Plein S (2016). Cardiac T1 mapping and extracellular volume (ECV) in clinical practice: a comprehensive review. J Cardiovasc Magn Reson.

[15] Mouquet F, Lions C, de Groote P, Mouquet F, Lions C, de Groote P (2008). Characterisation of peripartum cardiomyopathy by cardiac magnetic resonance imaging. Eur Radiol.

[16] Renz DM, Röttgen R, Habedank D, Wagner M, Böttcher J, Pfeil A (2011). New insights into peripartum cardiomyopathy using cardiac magnetic resonance imaging. Rofo.

[17] Arora NP, Mohamad T, Mahajan N, Danrad R, Kottam A, Li T (2014). Cardiac magnetic resonance imaging in peripartum cardiomyopathy. Am J Med Sci.

[18] Haghikia A, Röntgen P, Vogel-Claussen J, Schwab J, Westenfeld R, Ehlermann P (2015). Prognostic implication of right ventricular involvement in peripartum cardiomyopathy: a cardiovascular magnetic resonance study. ESC Heart Fail.

[19] Schelbert EB, Elkayam U, Cooper LT, Givertz MM, Alexis JD, Briller J, et al. Investigations of Pregnancy Associated Cardiomyopathy (IPAC) investigators. Myocardial damage detected by late gadolinium enhancement cardiac magnetic resonance is uncommon in peripartum cardiomyopathy. J Am Heart Assoc. 2017;6. 10.1161/JAHA.117.005472. 10.1161/JAHA.117.005472 PMC5533034 28373243

[20] Pritchard JA (1965). Changes in blood volume during pregnancy. Anesthesiology.

[21] Cong J, Yang X, Zhang N, Shen J, Fan T, Zhang Z (2015). Quantitative analysis of left atrial volume and function during normotensive and preeclamptic pregnancy: a real-time three-dimensional echocardiography study. Int J Cardiovasc Imaging.

[22] Estensen ME, Beitnes JO, Grindheim G, Aaberge L, Smiseth OA, Henriksen T (2013). Altered maternal left ventricular contractility and function during normal pregnancy. Ultrasound Obstet Gynecol.

[23] Simmons LA, Gillin AG, Jeremy RW (2002). Structural and functional changes in left ventricle during normotensive and preeclamptic pregnancy. Am J Physiol Heart Circ Physiol.

[24] Li J, Umar S, Amjedi M, Iorga A, Sharma S, Nadadur RD (2012). New frontiers in heart hypertrophy during pregnancy. Am J Cardiovasc Dis.

[25] Messroghli DR, Radjenovic A, Kozerke S, Higgins DM, Sivananthan MU, Ridgway JP (2004). Modified look-locker inversion recovery (MOLLI) for high-resolution T1 mapping of the heart. Magn Reson Med.

[26] Vinnakota KC, Bassingthwaighte JB (2004). Myocardial density and composition: a basis for calculating intracellular metabolite concentrations. Am J Physiol Heart Circ Physiol.

[27] Taylor AJ, Salerno M, Dharmakumar R, Jerosch-Herold M (2016). T1 mapping: basic techniques and clinical applications. JACC Cardiovasc Imaging.

[28] Guilford JP (1956). Fundamental statistics in psychology and education.

[29] Eghbali M, Deva R, Alioua A, Minosyan TY, Ruan H, Wang Y (2005). Molecular and functional signature of heart hypertrophy during pregnancy. Circ Res.

[30] McDiarmid AK, Swoboda PP, Erhayiem B, Lancaster RE, Lyall GK, Broadbent DA (2016). Athletic cardiac adaptation in males is a consequence of elevated myocyte mass. Circ Cardiovasc Imaging.

[31] Sado DM, White SK, Piechnik SK, Banypersad SM, Treibel T, Captur G (2013). Identification and assessment of Anderson-Fabry disease by cardiovascular magnetic resonance noncontrast myocardial T1 mapping. Circ Cardiovasc Imaging.

[32] Shah AM, Pfeffer MA (2012). The many faces of heart failure with preserved ejection fraction. Nat Rev Cardiol.

[33] Pica S, Sado DM, Maestrini V, Fontana M, White SK, Treibel T (2014). Reproducibility of native myocardial T1 mapping in the assessment of Fabry disease and its role in early detection of cardiac involvement by cardiovascular magnetic resonance. J Cardiovasc Magn Reson.

[34] Hayashi T, Yamada S, Iwano H, Nakabachi M, Sakakibara M, Okada K (2016). Left ventricular global strain for estimating relaxation and filling pressure - a multicenter study. Circ J.

[35] Woodiwiss AJ, Libhaber CD, Majane OH, Libhaber E, Maseko M, Norton GR (2008). Obesity promotes left ventricular concentric rather than eccentric geometric remodeling and hypertrophy independent of blood pressure. Am J Hypertens.

[36] Savu O, Jurcuţ R, Giuşcă S, van Mieghem T, Gussi I, Popescu BA (2012). Morphological and functional adaptation of the maternal heart during pregnancy. Circ Cardiovasc Imaging.

[37] Sanghavi M, Rutherford JD (2014). Cardiovascular physiology of pregnancy. Circulation.

[38] Melchiorre K, Sharma R, Khalil A, Thilaganathan B (2016). Maternal cardiovascular function in normal pregnancy: evidence of maladaptation to chronic volume overload. Hypertension.

[39] Goland S, Modi K, Bitar F, Janmohamed M, Mirocha JM, Czer LS (2009). Clinical profile and predictors of complications in peripartum cardiomyopathy. J Card Fail.

[40] Thomsen HS, Morcos SK, Almén T, Bellin M-F, Bertolotto M, Bongartz G (2013). Nephrogenic systemic fibrosis and gadolinium-based contrast media: updated ESUR contrast medium safety committee guidelines. Eur Radiol.

[41] Chang SA, Choe YH, Jang SY, Kim SM, Lee SC, Oh JK (2012). Assessment of left and right ventricular parameters in healthy Korean volunteers using cardiac magnetic resonance imaging: change in ventricular volume and function based on age, gender and body surface area. Int J Cardiovasc Imaging.

[42] Le TT, Tan RS, De Deyn M, Goh EP, Han Y, Leong BR (2016). Cardiovascular magnetic resonance reference ranges for the heart and aorta in Chinese at 3T. J Cardiovasc Magn Reson.

